# MiR-96-5p Suppresses Progression of Arsenite-Induced Human Keratinocyte Proliferation and Malignant Transformation by Targeting Denticleless E3 Ubiquitin Protein Ligase Homolog

**DOI:** 10.3390/toxics11120978

**Published:** 2023-12-01

**Authors:** Yan Li, Qiaoshi Zhao, Jinyin Yao, Chunpeng Lv, Yanhui Gao, Dianjun Sun, Yanmei Yang

**Affiliations:** 1 Center for Endemic Disease Control, Chinese Center for Disease Control and Prevention, Harbin Medical University, Harbin 150081, China; 2 Key Lab of Etiology and Epidemiology, Education Bureau of Heilongjiang Province & Ministry of Health (23618504), Harbin Medical University, Harbin 150081, China; 3 Heilongjiang Provincial Key Laboratory of Trace Elements and Human Health, Harbin Medical University, Harbin 150081, China; 4 Institution of Environmentally Related Diseases, Harbin Medical University, Harbin 150081, China

**Keywords:** arsenite, miR-96-5p, DTL, cell proliferation, malignant transformation, xenograft model

## Abstract

Long-term exposure to arsenic has been linked to a variety of cancers, among which skin cancer is the most prevalent form. However, the mechanism underlying arsenic carcinogenesis is unclear, and there is still limited information on the role of miRNAs in arsenic-induced skin cancer. This study aims to explore the role of miR-96-5p in the arsenite-induced proliferation and malignant transformation of human HaCaT keratinocytes. The GEO database (accession numbers GSE97303, GSE97305, and GSE97306) was used to extract mRNA and miRNA expression profiles of HaCaT cells treated with or without 0.1 μmol/L sodium arsenite for 3 and 7 weeks. In this paper, according to the CCK8 assay result, HaCaT cells exposed to 0.1 μmol/L sodium arsenite for 48 h were finalized. CCK8, MTT, EdU incorporation, and colony formation assays were used to determine the viability and proliferation of HaCaT cells and transformed HaCaT (T-HaCaT) cells. The subcellular localization and relative expression levels of DTL, as well as miR-96-5p in HaCaT cells induced by arsenite, were determined via immunofluorescence, RT-qPCR, and Western blot. Dual-luciferase reporter assay was performed to identify miR-96-5p bound directly to DTL. Transfection of miR-96-5p mimics or DTL siRNA was conducted to verify the arsenite-induced viability of HaCaT cells and T-HaCaT cells. T-HaCaT cells and nude mice were used to construct arsenite-induced malignant transformation and an in vivo xenograft model to demonstrate the over-expressed effect of miR-96-5p. The results showed that DTL was the target gene of miR-96-5p. Meanwhile, we also found that 0.1 μmol/L sodium arsenite upregulated DTL by decreasing the miR-96-5p level, leading to the proliferation and malignant transformation of HaCaT cells. MiR-96-5p agomir treatment slowed the growth of transplanted HaCaT cells transformed by arsenite in a manner associated with DTL downregulation in the nude mice xenograft model. Taken together, we confirmed that miR-96-5p, as a potent regulator of DTL, suppressed arsenite-induced HaCaT cell proliferation and malignant transformation, which might provide a novel therapeutic target for the treatment of arsenic-induced skin cancer.

## 1. Introduction

Arsenic is a native metalloid element found in low concentration in virtually every component of the environment [1]. From both natural and anthropogenic sources, arsenic is mainly transported in water, resulting in water arsenic pollution. The World Health Organization (WHO) has stated that the maximum permissible concentration of arsenic in drinking water is 10 μg/L [2]. Nevertheless, elevated levels of arsenic have been detected in groundwater in over 70 nations spread across five continents, including Bangladesh, India, China, Chile, Argentina, North America, Ghana, and Romania, threatening over 200 million people [1,3,4]. Chronic exposure to low-dose inorganic arsenic leads to deleterious effects in multiple organs and tissues [5]. A serious risk to worldwide public health is drinking water exposed to inorganic arsenic [6]. Chronic arsenic exposure can cause skin lesions such as skin pigmentation and hyperkeratosis (abnormal thickening of the skin) [7,8]. These unfavorable lesions can be precursors to several forms of skin cancer, the most common type of cancer caused by arsenic exposure. Regretfully, those patients suffering from arsenic lack proven, efficacious therapeutic approaches. For this reason, research into the pathophysiology of arsenic-induced skin cancer is essential in order to establish a theoretical foundation for its prevention and therapy.

Abnormal gene transcription has been proposed to address arsenic toxicity and carcinogenicity [9]. Epigenetic alterations, including differential microRNA (miRNA) expression, have been reported to influence the pathogenesis caused by arsenic via altering some gene expression [10,11,12]. MicroRNAs (miRNAs) are endogenous, small, non-coding RNAs consisting of 19–25 nucleotides. They target and inhibit the expression of many genes at the post-transcriptional or translational levels, thereby regulating a wide array of biological and pathogenic processes, including carcinogenesis or malignant transformation of cells. In various cancer cells or malignant transformed cells induced by carcinogens, miRNAs have been proven to be heavily dysregulated. Depending on certain contexts, miRNAs might function as either oncogenes or tumor suppressors. It is reported that dysregulated miRNAs influence carcinogenesis and development via disturbing cell growth, differentiation, cell cycle, and cell death; therefore, miRNAs, as an inherent gene regulator, might be a promising predictive and therapeutic target [13]. Recently, miRNAs have been found to be an important regulator in the DNA damage response. It is known that arsenic is responsible for ubiquitous damage to mammalian tissues by disrupting chromosomes and damaging DNA strands [14,15,16]. Accumulating evidence has demonstrated the significance of miRNA-mediated gene regulation in the arsenite-induced malignant transformation of HaCaT cells [11,17,18], derived from normal adult skin keratinocytes, suggesting abnormal miRNA expression might contribute to the arsenite-induced malignant transformation. Therefore, it is worthy to further explore which miRNAs and their target genes mediate the carcinogenesis of arsenic.

Al-Eryani, L. et al. found that the alteration in miRNA expression caused by chronic exposure to arsenite would reveal early steps in the malignant transformation when they detected the miRNA and mRNA expression in HaCaT cells treated with 0.1 μmol/L sodium arsenite for 3 and 7 weeks using Affymetrix microarrays [19,20]. We discovered that miR-96-5p was decreased, but DTL was increased in sodium arsenite-treated HaCaT cells using Al-Eryani, L. et al.’s database (accession numbers GSE97303, GSE97305, and GSE97306) [19,20]. Furthermore, according to the public database TargetScan 7.2, DTL contains a direct binding site for miR-96-5p, suggesting that it may be a target gene of miR-96-5p. Denticleless E3 ubiquitin protein ligase homolog (DTL), a critical regulator of chromosome segregation, DNA replication, and cell division [21], is elevated in human gastric and breast cancers and various cell lines derived from these primary tumors [22,23]. MiR-96-5p, however, may act as a tumor suppressor or tumor promoter, depending on the context. Therefore, we inferred that miR-96-5p might be involved in arsenite-induced HaCaT cell proliferation and malignant transformation. In this study, we aim to investigate the function of miR-96-5p in arsenite-induced proliferation and malignant transformation of HaCaT cells. This research helps to illustrate the mechanism underlying arsenic carcinogenesis and to provide new therapeutic targets in arsenic-induced skin cancer.

## 2. Materials and Methods

### 2.1. Microarray Data Analysis

The mRNA and miRNA expression profiles in HaCaT cells treated with or without 0.1 μmol/L sodium arsenite for 3 and 7 weeks were extracted from the GEO database, accession numbers GSE97303, GSE97305, and GSE97306. The CEL files were imported into Partek Genomics Suite (Partek Inc., St. Louis, MO, USA), and the differentially expressed mRNA or miRNA were analyzed considering arsenite treatment and time. Subsequently, the statistical correlation between differentially expressed miRNA and mRNA was calculated by the Partek Genomics Suite.

### 2.2. Cell Culture and Treatment

The HaCaT cells, developed from normal adult skin keratinocytes [24], were purchased from the Procell Life Science & Technology Co., Ltd. (Wuhan, China). Cells were cultured in a DMEM medium (HyClone, Logan, UT, USA) supplemented with 10% fetal bovine serum (Gibco, Grand Island, NY, USA), 100 U/mL penicillin, and 100 mg/mL streptomycin (Beyotime, Shanghai, China) and kept in a humidified incubator at 37 °C with 5% CO_2_. The cells were exposed to 0 or 0.1 μmol/L sodium arsenite (NaAsO_2_, purity: 99.0%, Sigma, St Louis, MI, USA). For chronic exposure, 6 × 10^5^ HaCaT cells were maintained in 25 cm cell culture flasks in a medium containing 0.1 μmol/L sodium arsenite and were subcultured every 48–72 h for about 40 weeks (approximated to 80 passages).

### 2.3. Cell Transfection

MiR-96-5p mimics, DTL siRNA, and respective scramble control were purchased from Shanghai Jima Co. (Shanghai, China). HaCaT cells were equally distributed into a 6-well plate with 2 × 10^6^ cells per well and cultured for 24 h. Then, 30 pmol miR-96-5p mimics (5′-UUUGGCACUAGCACAUUUUUGCU-3′ and 5′-CAAAAAUGUGCUAGUGCCAAAUU-3′), DTL siRNA (5′-GCACAUACUUCCAUAGAAATT-3′ and 5′-UUUCUAUGGAAGUAUGUGCTT-3′), miR-96-5p scramble (5′-GGCCUAACACUACUAACGCTT-3′ and 5′-GCGUUAGUAGUGUUAGGCCTT-3′) or DTL con-siRNA (5′-UUUUCCGAACGUGUCACGUTT-3′ and 5′-ACGUGACACGUUCGGAGAATT-3′) were transfected into HaCaT cells using Lipofectamine RNAiMAX (Invitrogen, Waltham, MA, USA) according to the manufacturer’s protocol. After 6 h, HaCaT cells were treated with 0.1 μmol/L sodium arsenite for another 48 h. Then, the transfected cells were harvested for subsequent experiments after the transfection efficacy was verified via the RT-qPCR analysis.

### 2.4. Cell Viability Assay

The Cell Counting Kit-8 (CCK-8) assay was used to determine the effects of arsenite on HaCaT cell viability. HaCaT cells were seeded in 96-well plates (5 × 10^3^ cells/well) and incubated for 24 h. Then, cells were treated with different concentrations of sodium arsenite (0, 0.05, 0.1, 0.2, 0.4, 0.8, and 1.0 μmol/L) in a DMEM medium containing 10% FBS for 48 h. An amount of 10 μL of CCK-8 (Dojindo, Kumamoto, Japan) solution was added to each well. Plates were incubated for a further 1 h at 37 °C and optical densities values at 450 nm (OD_450_) were recorded using Cytation 3 Cell Imaging Reader (BioTek, Winooski, VT, USA). Cell viability was calculated according to the formula below:

$$Cell\;viability\;\left(\%\right)=\frac{{OD}_{450}\left[experimental\;group\right]-{OD}_{450}\left[blank\;group\right]}{{OD}_{450}\left[control\;group\right]-{OD}_{450}\left[blank\;group\right]}\times 100\%$$

### 2.5. EdU Incorporation Assay

Exposed to 0.1 μmol/L of sodium arsenite for 48 h, HaCaT cells were incubated with DMEM supplemented with 25 μmol/L 5-Ethynyl-2′-deoxyuridine (EdU, RiboBio, Guangzhou, China) for 2 h. Cells were then washed with phosphate-buffered saline (PBS), followed by 4% paraformaldehyde (PFA) fixation and incubation with glycine 2 mg/mL, washed with PBS twice, and permeabilized with 0.5% Triton X-100 (Biofroxx, Einhausen, Germany). After extensive washing with PBS, cells were incubated with Apollo^®^ staining solution for 30 min, washed with 0.5% Triton X-100 three times, followed by 10 min incubation with Hoechst 33342. Photographs of the cells were captured with a laser-scanning confocal microscope (Zeiss, Oberkochen, Germany).

### 2.6. Cell Cycle Assay

HaCaT cells were plated in 6-well plates at a density of 2 × 10^5^ cells per well and were allowed to adhere overnight in a complete medium. Then, cells were starved for 12 h in a DMEM complete medium containing 2% FBS. Cells were harvested at 48 h after exposure to 0.1 μmol/L of sodium arsenite. After that, cells were fixed in 70% ice-cold ethanol overnight at −20 °C. The fixed cells were then suspended in lysis buffer with 0.1% Triton X-100 and incubated for 15 min at room temperature. The cells were incubated with 10 mg/mL RNAse A (Sigma, St Louis, MI, USA) for 10 min at room temperature, and DNA was stained with 50 mg/mL propidium iodide (PI) for at least 30 min at 4 °C. PI-stained cells were examined for their DNA content, which was determined via flow cytometry using a BD Accuri^TM^ C6 Plus (Becton Dickinson, Franklin Lakes, NJ, USA) equipped with the ModiFit LT v5.0 software.

### 2.7. Immunofluorescence

The cells were fixed in cell fixative containing 4% PFA, permeabilized with 0.1% Triton X-100 for 30 min at room temperature, and were blocked with 1% BSA for 1 h. The cells were incubated with the diluted anti-DTL antibody (1:100, ab184548, Abcam, Cambridge, UK) for 1 h at room temperature. Following three repeat PBS washes, the cells were treated with secondary antibody in 1% BSA for 30 min at room temperature. Nuclei were stained with 10 μg/mL DAPI (Beyotime, Shanghai, China).

### 2.8. Cell Growth Curve

HaCaT and transformed HaCaT (T-HaCaT) cells were seeded in 96-well culture plates at a density of 1 × 10^3^ cells/well. Every day, 20 μL MTT (Biofroxx, Einhausen, Germany) solution (5 mg/mL) was added to each well, and the cells were then cultured in an incubator containing CO_2_ for 4 h. The culture solution was then removed, and 150 μL DMSO (Biofroxx, Einhausen, Germany) was added to each well; the plate was then agitated (MaxQ 4000, Thermo Scientific, Waltham, MA, USA) at room temperature for 8 min. The OD values of each well were measured using the Cytation 3 Cell Imaging Reader. Six wells of each cell line were monitored every 24 h for 1 week. Cell growth curves of the two cell lines were drawn, and doubling times were calculated.

### 2.9. Flat Plate Colony Formation Assay

HaCaT cells were seeded into six-well plates with a density of approximately 200 cells per well, transfected with miR-96-5p mimics or scramble, and were incubated at 37 °C for 14 days with a switch to fresh medium every 3 days. Cell colonies were then fixed with 4% PFA for 10 min at room temperature and stained with 0.05% crystal violet for 30 min. With the use of a gel documentation system (Tanon, Shanghai, China), the plates were photographed, and the colony numbers were counted.

### 2.10. Soft-Agar Colony Formation Assay

Soft agar dishes were prepared under layers of 0.70% agarose (Lonza, Basel, Switzerland) in a DMEM medium with 10% FBS added. Cells were plated in triplicate at a density of 1 × 10^3^ in 1 mL of 0.35% agarose over the agar base to assess colony growth capacity. The plates were incubated at 37 °C for 2 weeks with a change of fresh medium every 3 days; colonies with >50 cells were examined microscopically.

### 2.11. RT-qPCR

The expression of DTL mRNA and miR-96-5p was determined via RT-qPCR. After treatment with 0.1 μmol/L sodium arsenite for 48 h, cells were harvested, and total cellular RNAs were extracted using Trizol reagent (Invitrogen, Waltham, MA, USA) according to the manufacturer’s instructions. The subsequent operations were carried out as described previously [25]. Semi-quantification was calculated using the 2^−ΔΔCt^ method and normalized to β-actin or U6 expression for mRNAs or miRNAs. Specific primers were designed by Primer 5.0 software and were ordered from Shanghai ShengGong Co. (Shanghai, China). The primer sequences used in our study were listed as follows: miR-96-5p stem-loop primer: GTCGTATCCAGTGCAGGGTCCGAGGTATTCGCACTGGATACGACAGCAAA; miR-96-5p (5′-GCGTTTGGCACTAGCACATT-3′ and 5′-ACGCTTCACGAATTTGCGTGTC-3′); U6 (5′-CTCGCTTCGGCAGCACATATACT-3′ and 5′-ACGCTTCACGAATTTGCGTGTC-3′); DTL (5′-TAAAAGCTGGTGAGCTGATTGG-3′ and 5′-TCTTCCACCCGTACAGAATACA-3′), β-actin (5′-GAGCACAGAGCCTCGCCTTT-3′ and 5′-ACATGCCGGAGCCGTTGTC-3′). RT-qPCR was conducted in triplicate for each sample.

### 2.12. Luciferase Reporter Assay

Using TargetScan 7.2 software, DTL was predicted to be a target gene of miR-96-5p. The full-length 3′ untranslated region (UTR) of DTL was amplified by PCR and cloned at the SacI and XhoI sites into a luciferase reporter vector (Promega, Madison, WI, USA). The mutant or wild-type construct of DTL 3′UTR was constructed by RiboBio (RiboBio Co., Guangzhou, China). HEK293T cells in 96-well plates were co-transfected with a reporter construct (pmiR-null Report plasmid, pmiR-DTL 3′UTR-wt, pmiR-DTL 3′UTR-mut) and miR-96-5p mimics or miR-negative control (NC) using Lipofectamine 3000 reagent (Invitrogen, Waltham, MA, USA). A dual-luciferase reporter assay system (Promega, Madison, WI, USA) was used to measure the levels of luciferase activity after 48 h. The results were normalized by dividing the firefly luciferase activity by the Renilla luciferase activity in accordance with the manufacturer’s instructions.

### 2.13. In Vivo Tumor Model

The study was authorized by the Institutional Animal Ethical Committee of Harbin Medical University and maintained in a sterile environment according to standardized animal care guidelines. Male BABL/C nude mice, 4 to 6 weeks of age, were purchased from Beijing Vital River Laboratory Animal Technology Co., Ltd. (Beijing, China) in two batches (*n* = 12 and *n* = 18, respectively).

To check whether T-HaCaT cells acquired a malignant phenotype, 12 mice were randomly divided into two groups; approximately 5 × 10^6^ HaCaT or T-HaCaT cells were inoculated subcutaneously into the right flanks of BALB/c nude mice. Tumor development was checked using an electronic caliper every other day for measurements of length (L) and width (W) and calculated as follows: volume = L × W^2^/2. The mice were euthanized immediately when the tumor volume had grown to 1500 mm^3^.

To verify the tumor suppressor ability of miR-96-5p, 18 mice were inoculated subcutaneously with 5 × 10^6^ T-HaCaT cells. When the average volume of tumors reached 100 mm^3^, 18 mice were randomly divided into three groups and were treated every three days by tail vein injection with nuclease-free water (control group), miR-96-5p scramble (5′-UUUUCCGAACGUGUCACGUTT-3′ and 5′-ACGUGACACGUUCGGAGAATT-3′), or miR-96-5p agomir (5′-UUUGGCACUAGCACAUUUUUGCU-3′ and 5′-CAAAAAUGUGCUAGUGCCAAAUU-3′), respectively. Tumor development was assessed by measuring the length (L) and width (W) of the tumor every second day with an electronic caliper and calculated as follows: volume = L × W^2^/2. When the tumor volume of control mice grew to 1500 mm^3^, all the mice were euthanized, and tumors were excised, photographed, and fixed in formalin or frozen at −80 °C for further analysis.

### 2.14. Western Blot

HaCaT cells (transplanted tumor) were collected and homogenized in a lysis buffer containing proteinase inhibitors (Beyotime, Shanghai, China). Total protein concentration was measured using a BCA assay kit (Beyotime, Shanghai, China). The subsequent procedures were described previously [25]. Finally, the protein expressions were visualized and analyzed using ImageJ software. The primary antibodies used were as follows: DTL (1:1000, ab184548, Abcam, Cambridge, UK); β-actin (1:1000, #7074, CST, Danvers, MA, USA); and β-tubulin (1:1000, #2128, CST, Danvers, MA, USA).

### 2.15. Statistical Analysis

All experiments were repeated at least three times. Data are reported as mean ± standard deviation. The statistical significance between the two groups was assessed using Student’s 2-tailed *t*-test. One-way ANOVA followed by a Bonferroni-Dunn test was used for the comparison of more than two groups. A *p*-value of <0.05 was considered statistically significant.

## 3. Results

### 3.1. Effects of a Low Level of Arsenite on HaCaT Cells Viability and Proliferation

**Figure 1:**
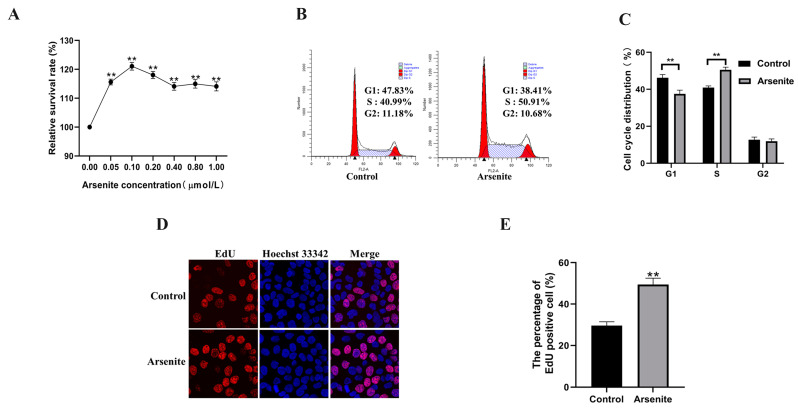
Effects of a low level of arsenite on the viability and proliferation of HaCaT cells. (**A**) HaCaT cells were treated with a gradient concentration of sodium arsenite for 48 h. Cell viability was evaluated by CCK-8 assays. (**B**) Flow cytometry was performed to analyze the cell cycle, and (**C**) statistical analysis of the cell cycle was displayed. (**D**) EdU incorporation assay of arsenite-exposed HaCaT cell groups and control cells. (**E**) Statistical analysis of EdU positive cells. Data were presented as means ± SD, *n* = 3, ** *p* < 0.01 vs. Control.

After gradient concentration (0.0, 0.05, 0.1, 0.2, 0.4, 0.8, and 1.0 μmol/L) of sodium arsenite treatments for 48 h, the viability of HaCaT cells was measured via WST-8 hydrolysis assay using a Cell Counting Kit-8 (Figure 1A). Compared with the control group, the sodium arsenite concentration interval 0.05 μmol/L to 1.0 μmol/L promoted HaCaT cells viability, and there was a proliferation peak value, 121.08 ± 1.33% when incubating with 0.1 μmol/L sodium arsenite (*p* < 0.01). Therefore, 0.1 μmol/L sodium arsenite treatment for 48 h was finalized in the following experiments. Subsequently, cell cycle distribution was analyzed via flow cytometry. As Figure 1B,C showed, these cell populations had a significant reduction in the proportion of G1 phase cells and a concomitant increase in the amount of S phase cells relative to control cells (*p* < 0.01), suggesting that 0.1 μmol/L sodium arsenite-induced an accelerated cell cycle transition from G1 to S phase. The EdU positive cell rate of 0.1 μmol/L arsenite-exposed cells was significantly increased compared with control cells (*p* < 0.01, Figure 1D,E). These results validated that 0.1 μmol/L sodium arsenite significantly increased keratinocyte HaCaT cell viability and proliferation.

### 3.2. Arsenite-Induced Malignant Transformation Model of HaCaT Cells Was Successfully Constructed

**Figure 2:**
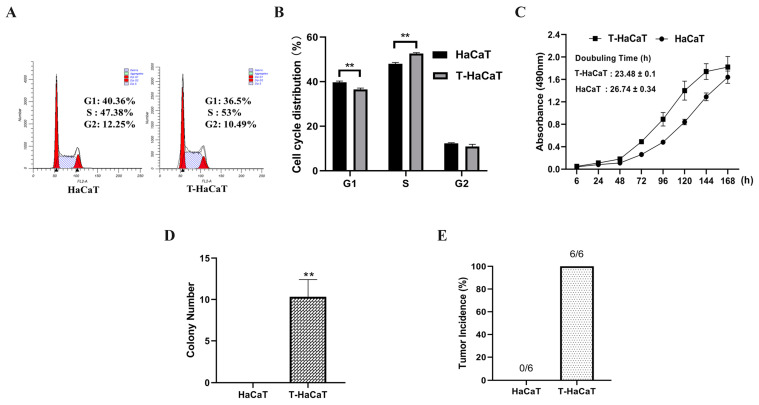
Arsenite induced malignant transformation model of HaCaT cells was successfully constructed. (**A**) Cell cycle, and (**B**) statistical analysis of T-HaCaT cells and passage control cells. (**C**) Growth curves in T-HaCaT cells and passage control cells. (**D**) The number of cell colonies in T-HaCaT cells and passage control cells. (**E**) T-HaCaT cells group and passage control group tumor incidences were displayed. Data were presented as means ± SD, *n* = 3, ** *p* < 0.01.

To construct the model of malignant transformation, HaCaT cells were exposed to 0.1 μmol/L sodium arsenite for about 40 weeks (approximated to 80 passages) and designated as T-HaCaT cells. Flow cytometry analysis indicated that the proportion of S phase in T-HaCaT cells was higher than that in passage control HaCaT cells (52.64 ± 0.41% vs. 48.03 ± 0.61%; Figure 2A,B). As Figure 2C shows, the doubling time of passage control HaCaT cells was 26.74 ± 0.34 h, and the value for T-HaCaT cells was 23.48 ± 0.10 h (*p* < 0.01). These results suggested that T-HaCaT cells presented a significant increase in cell proliferation compared with HaCaT cells.

Since transformed cells exhibit anchorage-independent growth [26], the evaluation of anchorage-independent growth of T-HaCaT cells was conducted. Two hundred T-HaCaT cells generated 10 ± 2 colonies, as shown in Figure 2D; in contrast, passage control HaCaT cells did not show anchorage-independent growth. Twelve male BALB/c-nude mice were used to examine tumorigenicity; in mice injected with T-HaCaT cells, tumor incidences were 100% (6 of 6), while tumor incidences for the control HaCaT cells were 0% (0 of 6; Figure 2E). As a result, it was possible to successfully construct the malignant transformation model of HaCaT cells triggered by 0.1 μmol/L sodium arsenite.

### 3.3. DTL Activation Involved in the Proliferation of HaCaT Cells Induced by Arsenite

**Figure 3:**
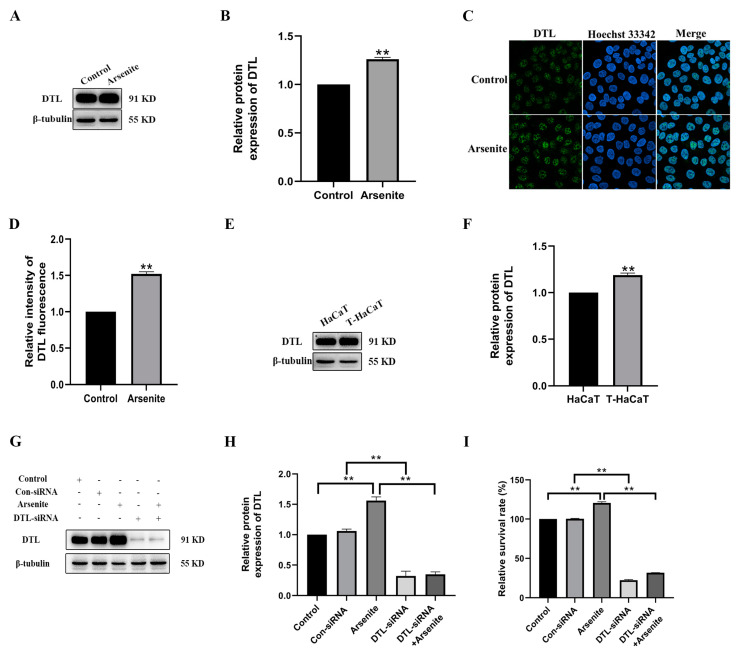
DTL activation mediated the proliferation of HaCaT cells induced by arsenite. (**A**) Protein levels of DTL in each group were presented by Western blot. β-tubulin was used as a loading control. (**B**) DTL relative protein expression levels were evaluated. (**C**) Immunofluorescence staining for DTL localization in HaCaT cells exposed to 0.1 μmol/L sodium arsenite. (**D**) The relative intensity of nuclear DTL fluorescence. (**E**) Protein levels of DTL in passage control cells and T-HaCaT cell groups were presented by Western blot. β-tubulin was used as a loading control. (**F**) DTL relative protein expression levels were evaluated. (**G**) HaCaT cells were transfected with DTL-siRNA or scramble control siRNA followed by 0.1 μmol/L sodium arsenite treatment for 48 h. DTL protein levels were displayed, and β-tubulin was used as a loading control. (**H**) DTL relative protein expression levels were evaluated, and (**I**) cell viabilities of all treatment groups were evaluated by CCK-8 assays. Data were presented as means ± SD, *n* = 3, ** *p* < 0.01.

Cell cycle regulation is the core part of cell proliferation, which has a close relationship with arsenic-induced carcinogenesis [27]. DTL is a master coordinator of cell cycle progression and is involved in carcinogenesis and development of various cancers [21,28]. In the microarray data extracted from the GEO database (accession numbers GSE97303, GSE97305, and GSE97306), the DTL mRNA in 0.1 μmol/L arsenite-treated HaCaT cells was upregulated to 1.57-fold (*p* = 0.28) and 3.06-fold (*p* = 0.015) at 3 and 7 weeks, respectively. Treatment with arsenite for 7 weeks increased the DTL mRNA to 2.19-fold (*p* = 0.016), compared with the 3-week arsenite-treated group. These results suggested that 0.1 μmol/L sodium arsenite treatment for several weeks can increase the DTL mRNA level. It is therefore intriguing to determine whether DTL protein is inducible in HaCaT cells exposed to arsenite. As shown in Figure 3A–D, the protein level of DTL in HaCaT cells exposed to arsenite for 48 h was increased. Meanwhile, 0.1 μmol/L sodium arsenite also increased the DTL level in the nucleus. In addition, the level of DTL protein also increased in the transformed T-HaCaT cells (Figure 3E,F). More importantly, while DTL expression was decreased effectively by specific siRNAs (Figure 3G,H), the arsenite-induced viability of HaCaT cells was dramatically reduced (Figure 3I). Thus, our findings indicated that DTL activation mediated the proliferation of HaCaT cells.

### 3.4. MiR-96-5p, a Negative Regulator of DTL, Mediated the Proliferation of HaCaT Cells Induced by Arsenite

Given the significance of DTL in proliferation and comparable overexpression of DTL in arsenite-exposed HaCaT cells, the exploration of mechanisms responsible for DTL expression seems extremely necessary. MicroRNAs regulate gene expression by repressing translation or inducing mRNA degradation at target sequences, which are often located in the 3′UTR. According to the microarray data extracted from the GEO database (accession numbers GSE97303, GSE97305, and GSE97306), miR-96-5p might be related to DTL, and miR-96-5p in arsenite-treated HaCaT cells was downregulated to 0.56-fold (*p* = 0.11) and 0.61-fold (*p* = 0.18) at 3 and 7 weeks, respectively. After arsenite treatment for 7 weeks, miR-96-5p in HaCaT cells was decreased to 0.58-fold (*p* = 0.047), compared with the 3-week arsenite-treated group. These data showed that miR-96-5p might be involved in the upregulation of DTL in response to 0.1 μmol/L sodium arsenite.

**Figure 4:**
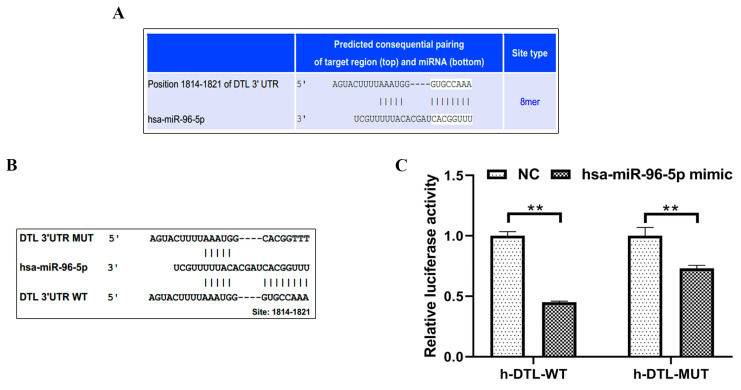
MiR-96-5p regulated DTL. (**A**) The schematic showed putative miR-96-5p target sites in the 3′UTR of DTL predicted by TargetScan 7.2. (**B**) Predicted miR-96-5p target site in the 3′UTR of DTL. The wild-type DTL mRNA sequence was shown with potential binding sites indicated at 1814-1821-bp. The highly conserved mature miR-96-5p sequence and potential binding between the miR-96-5p seed region to the DTL 3′UTR sequence were shown and mutated bases were indicated below. (**C**) MiR-96-5p mimics were cotransfected with the DTL wild-type or mutant 3′UTR as indicated. Luciferase activities were used to detect the effect on DTL expression at 48 h after transfection. Data were presented as means ± SD, *n* = 3, ** *p* < 0.01 vs. Control.

Using the TargetScan 7.2 (http://www.targetscan.org, accessed from 9 April 2018 to 22 April 2018), a direct binding site for miR-96-5p was predicted at 1814-1821-bp of DTL mRNA (Figure 4A). We created luciferase reporter vectors that contained the predicted DTL 3′UTR (3′UTR-wt) or mutant sequences (3′UTR-mut) in order to investigate the binding mechanism between miR-96-5p and DTL in more detail (Figure 4B). Notably, the greatest reduction in luciferase activity was observed in HEK293T cells after cotransfecting miR-96-5p mimics with DTL 3′UTR-wt (Figure 4C). When miR-96-5p mimics were cotransfected with DTL 3′UTR-mut instead of DTL 3′UTR-wt, the decline in luciferase activity was less pronounced, indicating the importance of these miR-96-5p binding sites in the DTL 3′UTR sequence for the function of miR-96-5p. Overall, the above findings revealed that miR-96-5p binds directly to the DTL-predicted sequence.

**Figure 5:**
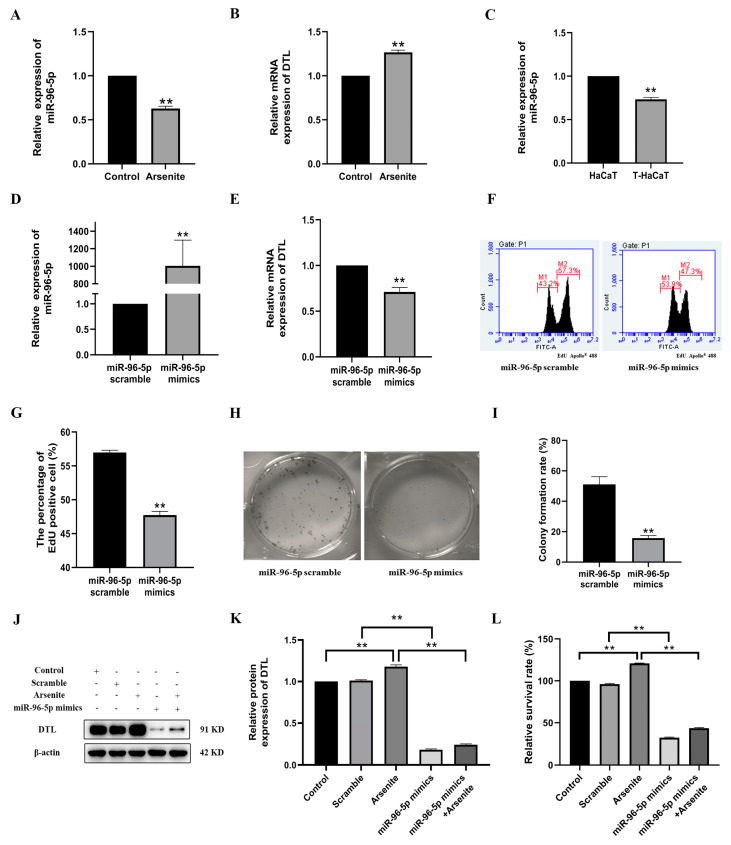
MiR-96-5p involved in the proliferation of HaCaT induced by arsenite. (**A**) The amount of miR-96-5p against U6 snRNA; (**B**) DTL mRNA against β-actin mRNA expression of arse-nite-exposed HaCaT cells and control cells were detected by RT-qPCR. (**C**) The amount of miR-96-5p against U6 snRNA in passage control cells and T-HaCaT cells. (**D**) HaCaT cells were transfected with miR-96-5p mimics compared to scramble control, the amount of miR-96-5p against U6 snRNA; (**E**) DTL mRNA against β-actin mRNA expression were detected by RT-qPCR. (**F**) EdU incorporation in HaCaT cells transfected with miR-96-5p scramble or miR-96-5p mimics was detected by flow cytometry. (**G**) Statistical analysis of EdU positive cells. (**H**) Colony formation in HaCaT cells transfected with miR-96-5p scramble or miR-96-5p mimics. (**I**) Colony formation rates were evaluated. (**J**) HaCaT cells were transfected with miR-96-5p mimics compared to scramble control followed by 0.1 μmol/L sodium arsenite treatment for 48 h. Protein levels of DTL and β-actin were presented. (**K**) DTL relative protein expression levels were evaluated. (**L**) Cell viability was evaluated by CCK-8 assays. Data were presented as means ± SD, *n* = 3, ** *p* < 0.01.

Upon investigating the regulatory role of miR-96-5p in tumorigenesis and its significant decrease in the cellular growth of colorectal cancer cells [29], we further explored the involvement of miR-96-5p in the proliferation of HaCaT cells exposed to sodium arsenite. MiR-96-5p was downregulated in sodium arsenite-treated cells (*p* < 0.01), compared with that in the control cells (Figure 5A,B). The level of miR-96-5p also decreased in the transformed T-HaCaT cells (Figure 5C). As shown in Figure 5D, when miR-96-5p mimics increased miR-96-5p level in HaCaT cells, the level of DTL mRNA in the miR-96-5p mimic-transfected cells reduced 29% (*p* < 0.01, Figure 5E), compared with cells transfected with the scramble, supporting the idea that miR-96-5p is capable of targeting the DTL 3′UTR to prevent DTL expression. Moreover, EdU staining measured via flow cytometry also demonstrated that miR-96-5p mimics substantially reduced the proliferation of HaCaT cells, compared with the scramble group (Figure 5F,G). In addition, a significant decrease in colony formation rates was also discovered in HaCaT cells overexpressing miR-96-5p (Figure 5H,I). Western blot assays also showed the enhancement of miR-96-5p attenuated the arsenite-induced increases in DTL expression (Figure 5J,K). Subsequently, cell viability analysis demonstrated the transfection of miR-96-5p mimics dramatically reduced the arsenite-induced viability of HaCaT cells (Figure 5L).

All of these findings together demonstrated unequivocally that miR-96-5p was involved in sodium arsenite-induced proliferation of HaCaT cells via negatively regulating DTL.

### 3.5. MiR-96-5p Treatment Suppressed T-HaCaT Cells Growth in Xenograft Model

**Figure 6:**
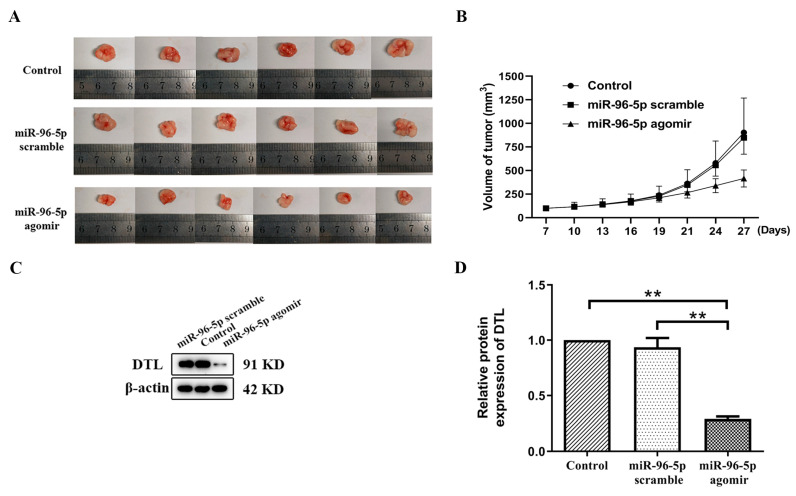
MiR-96-5p treatment suppressed T-HaCaT cell growth in the nude mice xenograft model. (**A**) The xenograft tumors were harvested from the nuclease-free water group, miR-96-5p scramble, and miR-96-5p agomir group. (**B**) Growth curves of xenograft tumors derived from different models were drawn. (**C**) DTL protein levels in xenograft tumor tissues were shown, β-actin was used as a loading control, and (**D**) DTL relative protein expression levels were calculated. Data were presented as means ± SD, *n* = 3, ** *p* < 0.01 vs. Control.

MiR-96-5p mimics may have the ability to reduce the proliferation of HaCaT cell growth, as revealed by in vitro studies. To further demonstrate the over-expressed effect of miR-96-5p in nude mice, we conducted an in vivo xenograft experiment with T-HaCaT cells. In total, 18 male nude mice were injected with T-HaCaT cells subcutaneously. When the average volume of tumors reached 100 mm^3^, 18 mice were randomly divided into three groups and were injected with nuclease-free water, miR-96-5p scramble, and miR-96-5p agomir, respectively. Three models exhibited differences on day 14 after the injection. The average tumor volume was significantly decreased in the miR-96-5p agomir treatment group compared with the other two groups (Figure 6A,B).

In addition, Western blot analysis showed that the miR-96-5p agomir group had a significantly lower level of DTL compared with the other two groups (Figure 6C,D). These in vivo and in vitro data concluded the negative regulation of DTL by miR-96-5p.

## 4. Discussion

Arsenic compounds have been recognized as anticancer drugs for the treatment of leukemia, including acute promyelocytic leukemia [30] and chronic myeloid leukemia [31] and inducing apoptosis in breast cancer cells [32] in clinical and in vitro trials. Nevertheless, as a known human carcinogen, chronic arsenic exposure results in skin pathology, including hyperkeratosis, pigmentation changes, Bowen’s disease, basal cell carcinomas, and squamous cell carcinomas [5,7]. Recent reports have indicated that chronic low-to-moderate arsenic exposure elevates the risk of urothelial tract cancers, for instance, bladder cancer and upper urothelial tract cancer [33,34]. In vitro studies also proved that chronic exposure to low concentrations of arsenic can induce malignant transformation of various cells, including HaCaT cells [35,36,37]. However, there is not yet agreement on the mechanisms underlying the arsenic-induced malignant transformation. It is well known that enhanced cell proliferation due to accelerated cell cycle progression may play key roles in the progression of malignant transformation induced by arsenite [38,39]. Low-dose arsenite can promote the proliferation of a variety of cells [12,40,41]. Our research showed that 0.1 μmol/L sodium arsenite, a concentration comparable to human blood arsenic levels found in chronic arsenicosis patients in Inner Mongolia, China [42], can induce malignant transformation of HaCaT cells. This concentration could increase the viability and proliferation of HaCaT cells, accompanied by an accelerated G1/S transition, manifested as the decrease in the proportion of G1 phase and an increase in the proportion of S phase. Therefore, it is worth studying the role of key molecular mediated in the cell cycle control in the proliferation and malignant transformation induced by arsenite.

In this study, we focused on the role of DTL in the proliferation and malignant transformation induced by arsenite. DTL is a critical gene for cell cycle regulation and DNA repair [21]. Error replication licensing and aberrant protein degradation are two possible initiators of genomic instability during carcinogenesis [43]. This is supported by previous data that the increased expression levels of DTL in aggressive hepatocellular carcinomas and colorectal cancer and that this expression level positively correlates with tumor progression and unfavorable prognosis [44,45,46,47]. DTL expression is also elevated in multiple myeloma, non-small cell lung cancer, ovarian cancer, head and neck squamous cell carcinoma, and cervical cancer [48,49,50,51,52,53]. In vitro, DTL can promote the growth of mammary epithelial cells, and silencing DTL via siRNA significantly impairs the growth of these cells with defects in apoptosis [22]. Although DTL has been investigated to be over-expressed in various cancers, its role in arsenic-induced skin cancer is unclear. Consistently, our study suggested that DTL mediated the proliferation of HaCaT cells induced by arsenite, which was approved by the following evidence: (1) DTL mRNA in arsenite-treated HaCaT cells was upregulated; (2) DTL protein expression was markedly increased in the HaCaT cells exposed to 0.1 μmol/L sodium arsenite for 48 h and arsenite-induced transformed T-HaCaT cells; and (3) knockdown of DTL by small interfering RNAs restrained the arsenite-induced proliferation of HaCaT cells. In conclusion, upregulated DTL might contribute to the proliferation induced by arsenite.

It is widely accepted that miRNAs, in most instances, fulfill their functions by binding to target genes and inhibiting the protein expression of these genes. Current studies have shown evidence that miRNAs regulate mRNAs in various physiological processes, including tumorigenesis [54]. Multiple studies have explored the role of miRNAs in the regulation of DTL. Li J. et al. declared that miR-490-5p inhibited the malignant progression of gastric cancer cells via DTL suppression [55]. Georges, S.A. et al. reported that miR-192 and miR-215 targeted DTL for degradation [56]. Baraniskin A. et al. found that miR-30a-5p was frequently downregulated in colon carcinoma and altered cell cycle via modulating DTL expression [57]. MiR-92 has been reported to regulate DTL by Helwak, A. et al. [58]. However, in the GEO database (accession numbers GSE97303, GSE97305, and GSE97306), none of these above-mentioned miRNAs were regulated in the HaCaT cells treated with arsenite for 3 and 7 weeks, while miR-96-5p was downregulated in the arsenite-treated HaCaT cells. We also found that miR-96-5p was downregulated in the HaCaT cells exposed to 0.1 μmol/L sodium arsenite for 48 h and arsenite-induced transformed T-HaCaT cells; moreover, the mRNA and protein level of DTL decreased as the level of miR-96-5p increased. Subsequently, the luciferase reporter assays revealed that the binding ability of miR-96-5p to the wild-type target sequence was stronger than that to the mutant target sequence, confirming that by specifically targeting the DTL 3′UTR, miR-96-5p regulates the expression of DTL. Therefore, DTL was the target and was regulated by miR-96-5p. Consequently, this finding partially corroborates our theory that miR-96-5p may be the main factor influencing DTL expression in skin cancer caused by arsenic exposure.

Due to conflicting data, it is unclear whether miR-96-5p promotes or suppresses tumor growth in cancer. A subgroup of human cancers, such as ovarian cancer [59], cervical cancer [60], hepatocellular carcinoma [61], gastric cancer [62,63], prostate cancer [64], and non-small cell lung carcinomas [65], have elevated expression of miR-96-5p, which causes key genes linked to cancer to be downregulated. On the other hand, miR-96-5p paradoxically suppresses proliferation in different cancer types, including osteosarcoma [66], pancreatic carcinoma [67], and colorectal cancer [29,68]. The function of miR-96-5p as a tumor anti-oncomiR is becoming progressively more unambiguous, although its influence on tumor suppression in skin cancer caused by arsenic exposure is unknown. In this work, we offer a comprehensive investigation into the role and likely underlying mechanisms of miR-96-5p in HaCaT cells.

Our investigation also revealed that miR-96-5p functioned as an anti-oncomiR, influencing the proliferation and progression of the cell cycle in HaCaT cells stimulated by arsenite. Firstly, we found that the miR-96-5p was decreased after treatment with 0.1 μmol/L sodium arsenite for 48 h in comparison with the unexposed group. Secondly, transfected miR-96-5p mimics reduced the growth of HaCaT cells induced by arsenite. Thirdly, in vivo tumor formation assays, the miR-96-5p agomir group displayed a smaller tumor volume than the miR-96-5p scramble group, enhancing the expression of miR-96-5p performed a positive function. The evidence presented above all pointed to miR-96-5p’s antitumorogenic function in the arsenite-induced carcinogenesis of HaCaT cells.

The following limitations remain in this work. Firstly, DTL acted as a mediator and was involved in the proliferation and malignant transformation of HaCaT cells caused by arsenite exposure, but direct evidence and precise molecular regulatory mechanisms need to be explored further. Additionally, the mechanism by which the miR-96-5p/DTL axis regulates its downstream signaling pathway in sodium arsenite-induced proliferation of HaCaT cells is yet to be fully elucidated.

## 5. Conclusions

In this study, we confirmed that 0.1 μmol/L sodium arsenite significantly decreased miR-96-5p levels and upregulated DTL, which in turn accelerated the cell cycle and led to malignant transformation. MiR-96-5p, in our study as an anti-oncomiR, inhibited the proliferation of HaCaT cells by targeting DTL. Furthermore, we found that miR-96-5p agomir treatment slowed tumor growth in a manner associated with DTL downregulation in the nude mice xenograft model. These findings uncover a potential oncogenic axis, the miR-96-5p/DTL axis, which might provide a novel therapeutic target for the treatment of arsenic-induced skin cancer.

## Data Availability

The data presented in this study are available on request from the corresponding author.
